# A hierarchical classification of adolescent idiopathic scoliosis: Identifying the distinguishing features in 3D spinal deformities

**DOI:** 10.1371/journal.pone.0213406

**Published:** 2019-03-20

**Authors:** Saba Pasha, Pedram Hassanzadeh, Malcolm Ecker, Victor Ho

**Affiliations:** 1 Perleman School of Medicine, University of Pennsylvania, Philadelphia, Pennsylvania, United States of America; 2 Division of Orthopedic Surgery, The Children's Hospital of Philadelphia, Philadelphia, Pennsylvania, United States of America; 3 Department of Mechanical Engineering, Rice University, Houston, Texas, United States of America; 4 Department of Radiology, University of Pennsylvania, Philadelphia, Pennsylvania, United States of America; 5 Department of Radiology, The Children's Hospital of Philadelphia, Philadelphia, Pennsylvania, United States of America; University of California San Francisco, UNITED STATES

## Abstract

This study aimed to identify the differentiating parameters of the spinal curves’ 2D projections through a hierarchical classification of the 3D spinal curve in adolescent idiopathic scoliosis (AIS). A total number of 103 right thoracic left lumbar pre-operative AIS patients were included retrospectively and consecutively. A total number of 20 non-scoliotic adolescents were included as the control group. All patients had biplanar X-rays and 3D reconstructions of the spine. The 3D spinal curve was calculated by interpolating the center of vertebrae and was isotropically normalized. A hierarchical classification of the *normalized* spinal curves was developed to group the patients based on the similarity of their 3D spinal curve. The spinal curves’ 2D projections and clinical spinal measurements in the three anatomical planes were then statistically compared between these groups and between the scoliotic subtypes and the non-scoliotic controls. A total of 5 patient groups of right thoracic left lumbar AIS patients were identified. The characteristics of the posterior-anterior and sagittal views of the spines were: **Type 1**: Normal sagittal profile and *S shape* axial view. T1 is leveled or tilted to the right in the posterior view. **Type 2:** Hypokyphotic and a *V shape* axial view. T1 is tilted to the left in the posterior view. **Type 3:** Hypokyphotic (only T5-T10) and frontal imbalance, *S shape* axial view. T1 is leveled or tilted to the right, and 3 frontal curves. **Type 4:** Flat sagittal profile (T1-L2), slight frontal imbalance with a *V shape* axial view, T1 tilted to the left. **Type 5:** flat sagittal profile and forward trunk shift with a proximal kyphosis and *S shape* axial view. T1 is leveled or tilted to the right. In conclusion, a hierarchical classification of the 3D scoliotic spine allowed identifying various distinguishing features of the spinal curves in patients with a right thoracic curve in an orderly fashion. The subtypes’ characteristics resulting from this 3D classification can be identified from the *pairs* of the frontal and sagittal spinal curves *i*.*e*. X-rays in right thoracic AIS patients.

## Introduction

Adolescent idiopathic scoliosis (AIS) is a three-dimensional (3D) deformity of the spinal column. Spinal surgery, both conventional fusion methods and growth modulating techniques [1, 2], remains the mainline treatment option for progressive spinal curves. The pre-operative spinal curve is shown to be an important factor in surgical decision-making [3, 4]. The application of the two-dimensional (2D) X-ray images for AIS diagnosis and classification has limited the characterization of the spinal curvature to 2D projections of the 3D spinal curvatures on orthogonal planes. Several limitations of such 2D measurements and classifications are shown [5, 6]. Methods for 3D classifications of the spinal curve in AIS have been explored [6–9], however, complicated and time consuming post-processing techniques associated with these classifications has hampered the dissemination of these classifications as readily applicable tools in clinical setups. To address the current limitation in the classification of AIS, a classification method that uses the 3D spinal curve to describe the differences in the frontal and sagittal spinal curvatures is of critical need.

The first widely used classification of the scoliotic spine in AIS focused on the frontal deformity of the curve [10]. Hyper-kyphosis, normal, and hypokyphosis sub-classifications were considered to incorporate possible sagittal modifiers in the frontal classification of AIS [3]. The axial plane rotation of the curve was shown to be a defining characteristic of the spinal curvature in AIS [11, 12]. Statistical clustering methods, which include both alignment and rotation of the vertebrae, have shown promising results in distinguishing between the curve patterns in AIS patients [6–9]. However, the application of the axial parameters in clinical assessment of the AIS patients remains limited [11, 13, 14].

Considering the importance of the 3D alignment of the spine on the postural balance and surgical outcome [15, 16], the current study aimed to develop a 3D classification of the spinal curve in right thoracic AIS patients, the most common scoliotic curve type. A hierarchical classification that allows identifying the differentiating features of the spinal curve in an orderly fashion was investigated. Orthogonal 2D projections of the 3D classification resulted from such classification was used to describe the differences between the 3D spinal curve patterns in the subgroups of right thoracic AIS patients.

## Materials and methods

### Subjects

A total number of 103 pre-operative AIS patients who had biplanar spinal X-rays were included retrospectively and consecutively. The study procedures was approved by the institutional review board (IRB) at the Children’s Hospital of Philadelphia. A waiver of consent/parental agreement was granted for the retrospective analysis of the de-identified data. Attention was paid to proper patient positioning and head alignment in all X-rays [17]. Age and sex were determined from the patient chart at the time of X-ray acquisition. All patients had a right thoracic and left thoracolumbar/lumbar curve identified with a thoracic apex at a disc or vertebral level between T2 and T11-T12 disc according to the Scoliosis Research Society terminology [18]. Exclusion criteria was prior spinal surgery, spinal abnormality other than scoliosis, and neuromuscular conditions. No criterion based on the curve severity was considered. Twenty non-scoliotic adolescents, verified by spinal X-rays and clinical examinations, were added as the control group. The AIS and control groups had 3D reconstructions of the spine and pelvis generated in SterEOS software (EOS imaging, Paris, France) [19].

### Clinical parameters

The 3D model of the spine was used to calculate the following clinical spinal parameters in SterEOS software to eliminate the errors associated with the 2D measurements [5, 20, 21]: Proximal thoracic Cobb (PTC), Proximal thoracic rotation (apical) (PTR), Main thoracic Cobb (MTC), Main thoracic rotation (apical) (MTR), Lumbar Cobb (LC), Lumbar rotation (apical) (LR), Thoracic kyphosis (TK) between T1-T4 and T4-T12 levels, L1-S1 lumbar lordosis (LL), Pelvic incidence (PI), Sacral slope (SS), Pelvic tilt (PT), and Pelvic obliquity (PO).

The 3D reconstruction of the spine was used to determine the T1-L5 vertebral centroids using a method described before [22]. In this method, the average 3D coordinate of the superior and inferior vertebral endplates centroids determined the vertebral body centroid [22]. Frontal balance (FB) and sagittal balance (SB) were measured as the horizontal distances between the T1 plumbline and the posterior aspect of sacrum in frontal and sagittal planes, respectively. A total number of 15 clinical parameters were measured in our analysis.

### 3D spinal curve and hierarchical classification

A 3D curve was defined by connecting the T1 to L5 vertebral centroids in MATLAB R2017a (The MathWorks Inc, Natick, MA)[16]. An isotropic scaling was used to normalize the spinal heights by multiplying the (X, Y, Z) coordinates of the spine in a unique number for each patient in a way that unit size in the Z direction is achieved. Finally, 17 points at equal Z-levels for each spine were determined using a linear interpolation of the 3D positions of the consecutive vertebral centroids. These 17 Z levels were determined at the T1 to L5 Z coordinates of the average normalized spines of the non-scoliotic cohort.

The (X, Y) coordinates of each normalized spine (patient) formed a column of a data matrix. An agglomerative hierarchical clustering algorithm in MATLAB R2017a (The MathWorks Inc, Natick, MA) was used to merge the most similar 3D spines into one cluster. This clustering algorithm minimized the variance within each cluster of spines while maximizing the differences between the clusters of spines (Ward’s method) [23]. A clustering tree resulting from this analysis merges the most similar patient(s) at multiple steps (Ward’s distances) until all patients are in one group (103 patients). The cutoff value to determine the maximum number of clusters (subgroups) was determined at the lowest Ward’s distance at which all the determined clusters were visually different. The clinical variables of the patients’ group were statistically compared using an analysis of variance each time two groups of patients were merged.

## Results

### Patient population

**Fig 1:**
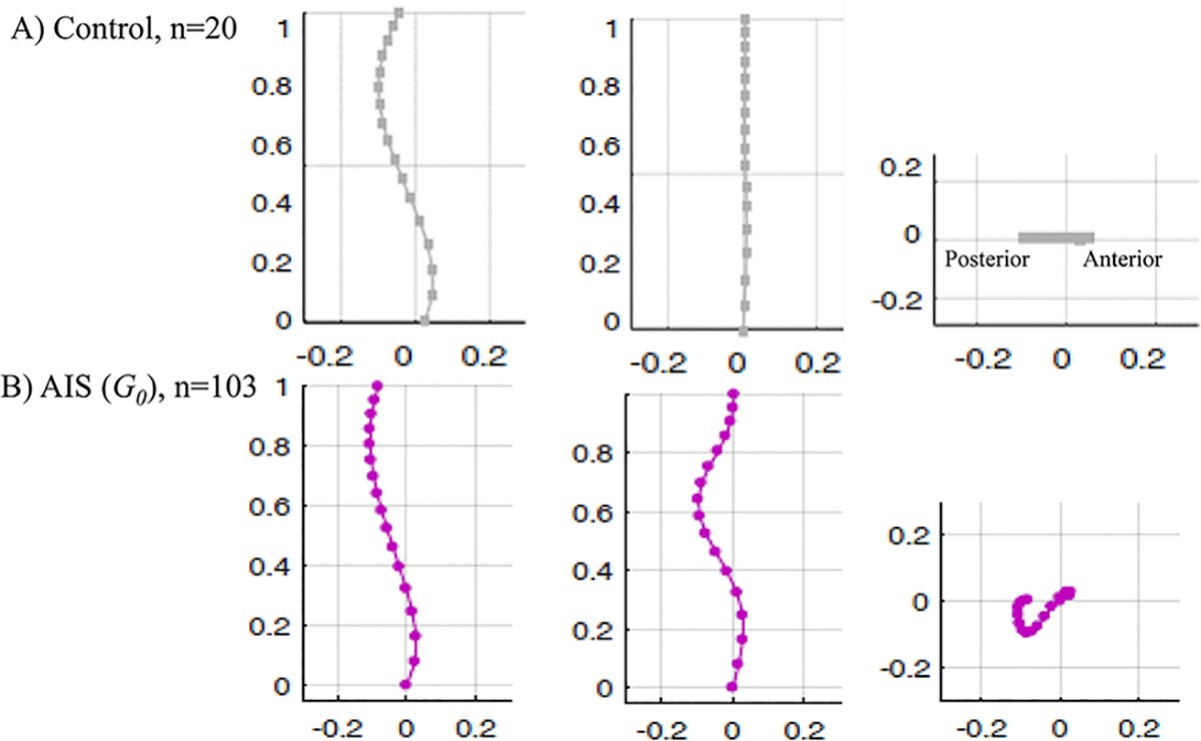
The average position of the vertebral centroids in A) the non-scoliotic control group (n = 20) and B) the AIS cohort (n = 103) in sagittal, frontal, and axial views are shown.

The average age at the time of radiography was 13±3 year. 85% of the patients were female. The average shape of the normalized spinal curve in the frontal, sagittal, and axial planes for all the AIS patients (n = 103) and the non-scoliotic cohort (n = 20) are shown in Fig 1.

### Cluster analysis

**Fig 2:**
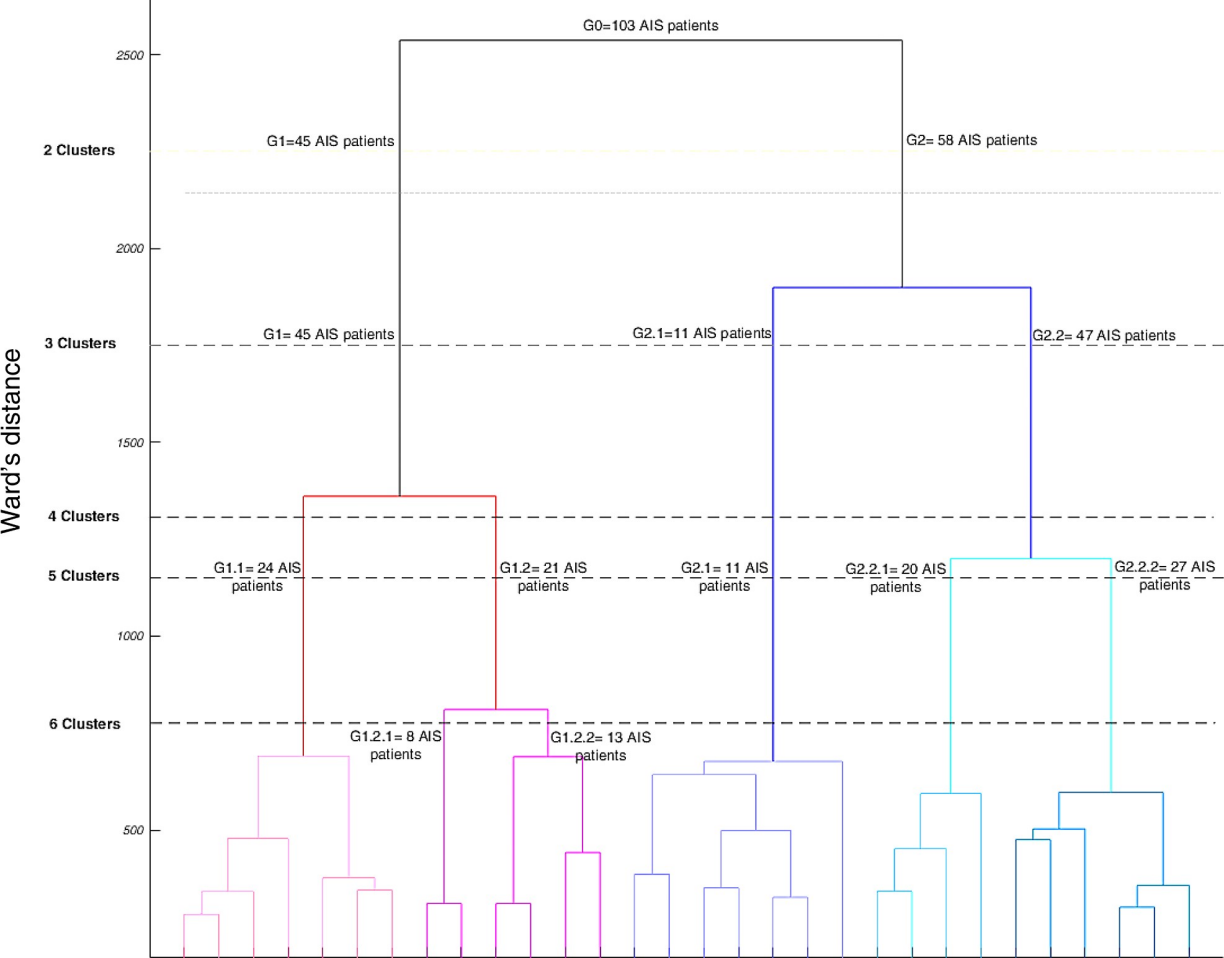
The dendrogram of the AIS patients hierarchical classification (n_0_ = 103). The Y-axis shows the Ward’s distances at which the increase in within cluster variance remains minimum after merging two groups. The number of patients in each cluster at different Ward’s distances are shown. At the highest cutoff point two clusters of G_1_ = 45 patients and G_2_ = 58 patients were identified. At Ward’s distance = 1700, two subgroups of G_2_, G_2.1_ = 11, G_2.2_ = 47 were identified. At Ward’s distance = 1320, two subgroups of G_1_ are shown (G_1.1_ = 24, G_1.2_ = 21). Finally, at Ward’s distance = 1125, G_2.2_ subgroups (G_2.2.1_ = 20, G_2.2.2_ = 27) were shown. The two subgroups of G_1.2_ (G_1.2.1_ = 8, G_1.2.2_ = 13), as shown at Ward’s distance = 790, were not visually different (supplementary data- S1 Fig).

**Fig 3:**
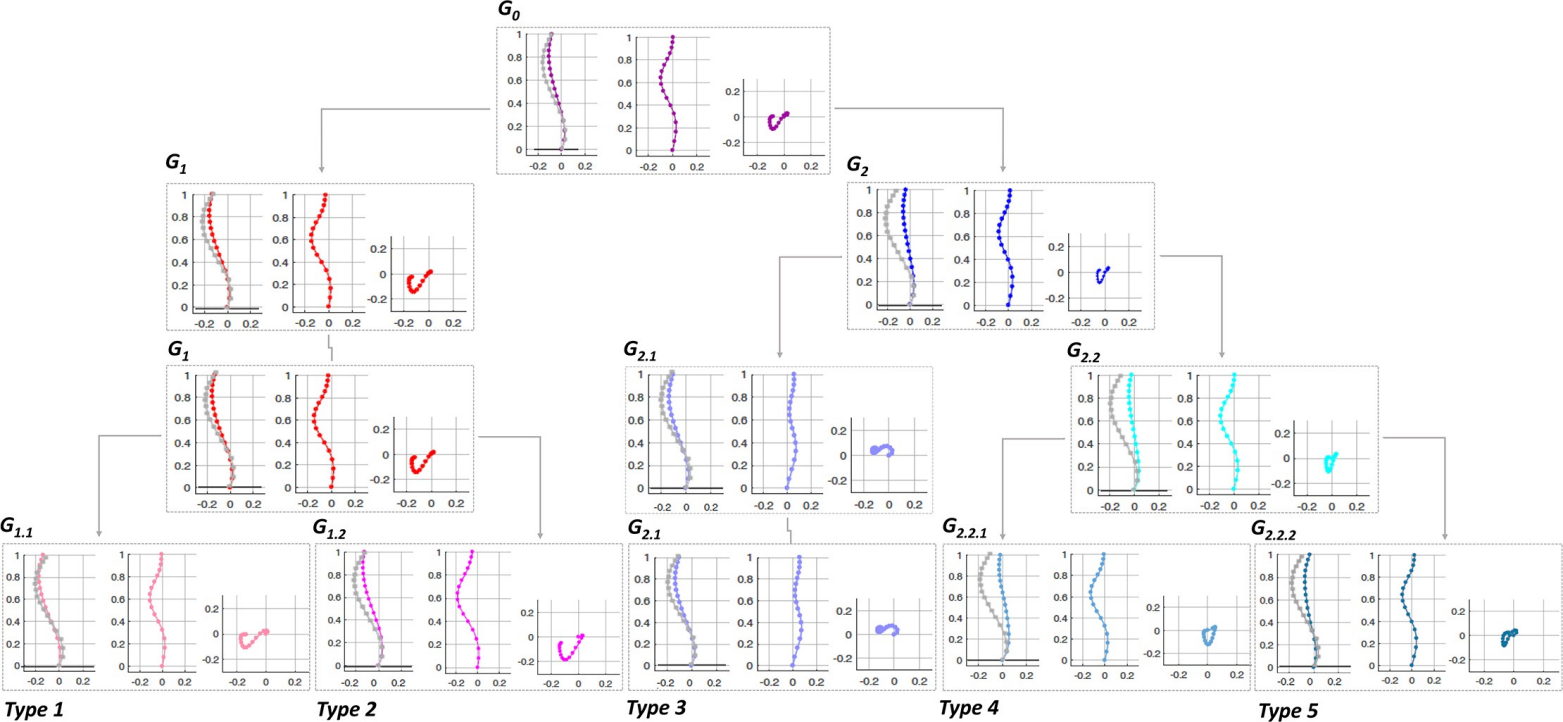
Sagittal, frontal, and axial views of the spine corresponding to the clusters in Fig 2. The colors and groups names matches the clusters in the dendrogram (Fig 2). The average of the non-scoliotic normalized sagittal spine is superimposed on the sagittal view of each AIS cluster for a visual comparison. The five subtypes (Type 1 to Type 5) are shown.

The dendrogram plot showing the clustering tree is presented in Fig 2. The number of patients and the Cutoff values (Ward’s distance) where the braches were merged are shown where 2 to 6 clusters were identified (Fig 2). Fig 3 shows the three anatomical views of the spine in each cluster as determined in the clustering tree (Fig 2).

Starting from the top of the clustering tree (Figs 2 and 3), the largest two clusters G_1_ (n = 45, 44%) with a normal/hypo-thoracolumbar kyphosis and G_2_ (n = 58, 56%) with a flat sagittal profile were identified. These two clusters differed in the sagittal profiles as also measured by the clinical variables, T4-T12 kyphosis and SB (Table 1), p<0.05. The axial view was different between the G_1_ and G_2_ clusters, showing larger posterior and lateral shifts of the spine in G_1_ compared to G_2_ cluster (Fig 3).

**Table 1:** Summary of clinical parameters in the clusters. PTC: proximal thoracic Cobb angle, PTR: proximal thoracic apical rotation, MTC: main thoracic Cobb angle, MTR: main thoracic apical rotation, LC: lumbar Cobb angle, LR: lumbar apical rotation, TK thoracic kyphosis, LL lumbar lordosis, PI: pelvic incidence, SS: sacral slope, PT, pelvic tilt, PO: pelvic obliquity, FB: frontal balance, SB: sagittal balance.

Cluster		PTC°	PTR°	MTC°	MTR°	LC°	LR°	TK° (T1-T4)	TK° (T4-T12)	LL° (L1-S1)	PI°	SS°	PT°	PO°	FB (mm)	SB (mm)
**All**	G_0_, N = 103	3.1±9.9	0.7±12.5	58.7±18.9	12.5±11.6	41.8±17.2	10.9±13.7	11.4±19.7	14.7±17.2	54.5±23.2	51.7±25.4	44.4±20.6	7.3±20.1	3.9±7.3	0.0±19.0	-4.1±12.3
**2 Clusters**	G_1_, N = 45	2.7±6.8	1.0±6.5	61.3±13.9	15.5±8.1	39.8±12.5	11.3±8.3	15.6±12.9	**18.8±8.2**	57.5±16.5	50.9±19.4	45.2±15.9	5.6±11.3	3.3±5.0	-12.5±11.3	**-10.4±9.4**
	G_2_, N = 58	3.3±7.8	0.5±10.6	56.8±12.8	10.1±8.5	43.3±11.6	10.5±10.9	8.7±11.8	**9.6±7.1**	52.1±15.1	52.4±16.4	43.7±13.2	8.7±16.7	4.4±5.4	9.7±15.3	**0.9±7.8**
**3 Clusters**	G_1_, N = 45	2.7±6.8	1.0±6.5	61.3±13.9	15.5±8.1	37.8±12.5	11.3±8.3	15.6±12.9	**18.8±8.2**	57.5±16.5	50.9±19.4	45.2±15.9	5.6±11.3	3.3±5	**-12.5±11.3**	**-10.4±9.4**
	G_2.1_, N = 11	5.2±3.3	1.6±5.9	58.9±7.5	11.7±5.4	44.2±7.5	11.6±5.4	9.6±8.2	12.6±5.4	51.5±7.8	48.9±11.2	43.1±7.4	5.1±8.5	5.1±4.2	**40.8±11.1**	-5.2±3.5
	G_2.2_, N = 47	2.9±7.3	0.3±8.9	56.3±10.5	9.8±6.4	42.1±8.8	10.2±9.5	8.3±12.3	**8.6±9.7**	52.3±13.9	53.2±11.9	43.8±10.9	9.5±14.3	4.3±3.6	**2.5±10.6**	**2.3±7.2**
**5 Clusters**	G_1.1_, N = 24	5.1±5.5	0±5.4	59.3±8.3	14.7±6.1	40.5±7.7	12.0±5.6	**16.7±7.**7	**21.7±6.5**	59.5±12.5	48.7±13.2	44.8±11.6	3.5±8.1	3.1±3.0	-9.9±8.1	**-11.1±8.0**
	G_1.2_, N = 21	0.0±4.1	2.1±3.7	**63.5±10.2**	**16.5±5.3**	38.3±9.8	10.6±6.1	14.4±9.6	15.4±7.0	55.2±11.3	53.5±14.3	45.7±10.9	7.9±7.9	3.5±4.1	**-15.5±8.4**	-9.6±4.7
	G_2.1_, N = 11	5.2±3.3	1.6±5.9	58.9±7.5	11.7±5.4	44.2±7.5	11.6±5.4	9.6±8.2	12.6±5.4	51.5±7.8	48.9±11.2	43.1±7.4	5.1±8.5	5.1±3.9	**40.8±10.5**	-5.2±3.5
	G_2.2.1_, N = 20	3.8±5.1	0.1±5.1	57.9±8.3	10.8±4.3	41.4±4.7	10.5±7.1	**8.1±6.4**	**8.5±6.1**	53.2±9.5	51.5±7.3	43.5±8.6	7.7±12.4	2.7±2.4	-10.8±8.1	**4.8±5.8**
	G_2.2.2_, N = 27	2.2±5.2	0.5±7.3	**54.3±6.4**	**9.3±4.8**	44.2±7.5	10.0±6.3	8.6±8.0	8.9±7.7	51.6±10.2	54.4±9.5	44.1±6.8	10.9±7.3	5.4±3.6	12.3±7.1	**0.4±4.3**
**Control**	N = 20	-	-	-	-	-	-	13.4±4.6	21.2±7.2	53.9±12.3	45.1±8.9	33.2±9.8	12.5±9.1	0±2.8	0±7.2	-14.8±4.8

Significant differences are shown in bold (*p*<0.05)

Moving down the clustering tree (Fig 2), three groups of patients were determined. These clusters are G1 (unchanged) and the two clusters that have been merged to create the G_2_ cluster: G_2.1_ and G_2.2_ (Fig 2). These two new clusters characteristics are (Fig 3): G_2.1_: hypokyphosis and positive frontal balance (shift to the left) and G_2.2_ with flat sagittal profile and balanced in the frontal plane. T4-T12 kyphosis was significantly different between G_1_ and G_2.2_, p<0.05. FB in G_2.1_ was significantly different from G_1_ and G_2.2_. SB was significantly different between the G_2.2_ and G_1_, p<0.05 (Table 1).

Description of 4 clusters was skipped since the clusters characterizations are included when either 3 or 5 clusters were described (Fig 3). When 5 groups were identified, G1 sub-clusters were: G_1.1_ (n = 24, 23%) and G_1.2_ (n = 21, 20%) (Fig 2) and G_2.2_ sub- groups were, G_2.2.1_ (n = 20, 19%) and G_2.2.2_ (n = 27, 26%). G_2.1_ remained unchanged (n = 11, 11%). G_1.1_ had a similar average sagittal curve to the non-scoliotic cohort and frontally balanced, G_1.2_ and G_2.1_ differed in sagittal profile particularly in the thoracolumbar region (Fig 3). G_2.2.1_ and G_2.2.2_ had a flat thoracic kyphosis and a forward trunk shift without and with a proximal kyphosis, respectively (Fig 3). The MTC and MTR were significantly higher in G_1.2_ compared to G_2.2.2_. TKs (both T1-T4 and T4-T12) were higher in G_1.1_ compared to G_2.2.1_. FB varied between G_1.2_ and G_2.1_ and SB between G_1.1_ and both G_2.2.1_ and G_2.2.2_, *p*<0.05 (Table 1). Comparing the axial views between the 5 clusters, clear differences between the curve types were observed; the apex of the thoracic curve was closer to the true frontal plane in G_1.2_, G_2.2.1_, and G_2.2.2_ and closer to the true sagittal plane in G_1.1_ and G_2.1_ (Fig 3). G_1.1_, G_2.1_, and G_2.2.2_ had S shaped axial projection of the spine whereas G_1.2_ and G_2.2.1_ had V shaped axial projection of the spine (Fig 3).

**Fig 4:**
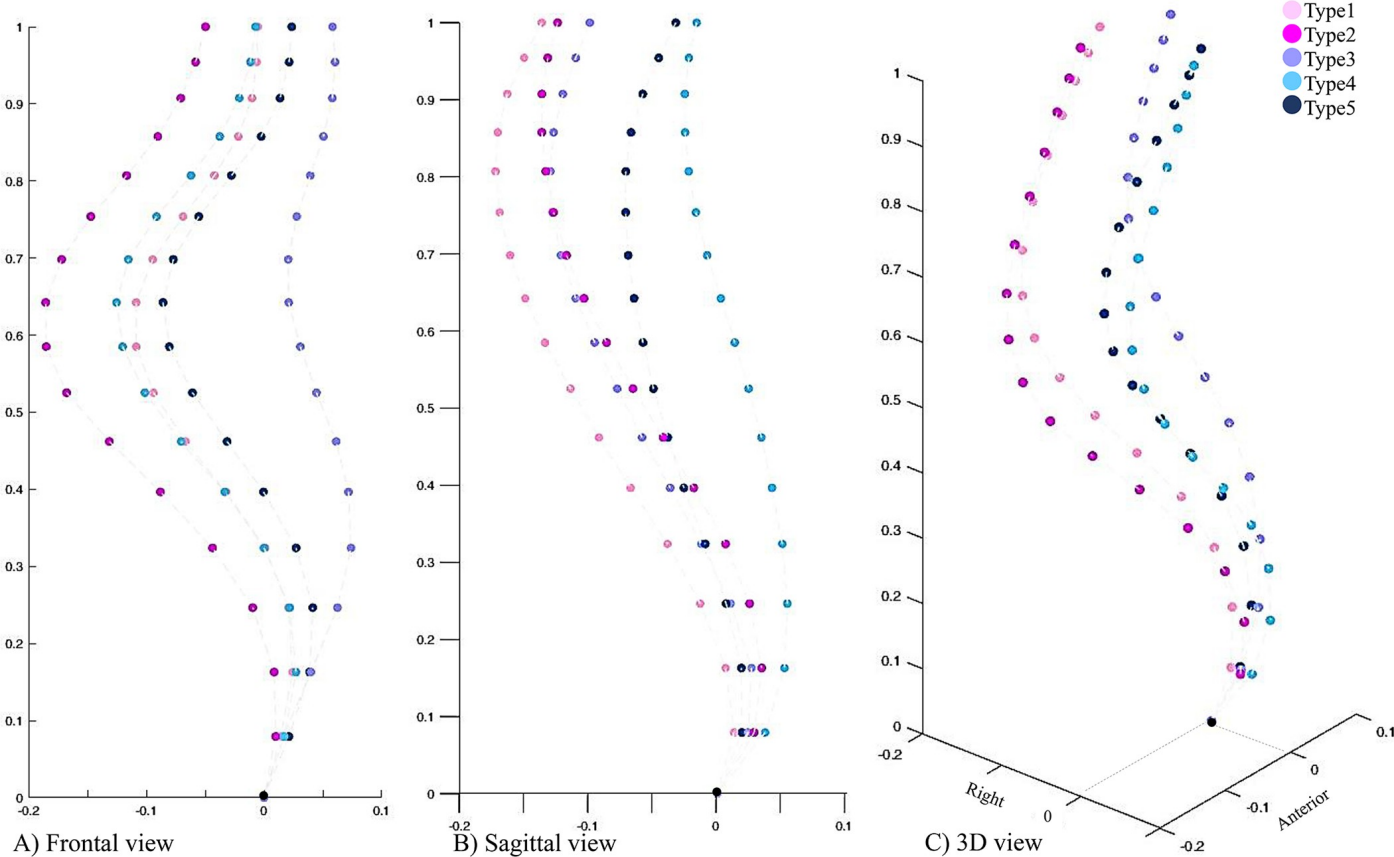
The final 5 clusters A) frontal, B) sagittal and C) 3D views are superimposed. Frontal view is comparable between G_1.1_, G_2.2.1_, G_2.2.2_ when compared to either G_1.2_ or G_2.1_ (Fig 4A). The sagittal profile was more comparable between G_1.1_, G_1.2_, G_2.1_ compared to either G_2.2.2_ or G_2.2.1_ (Fig 4B). In 3D, two main clusters (G_1_s and G_2_s) were identifiable (Fig 4C). The colors and groups names matches the clusters in Figs 2 and 3.

**Fig 5:**
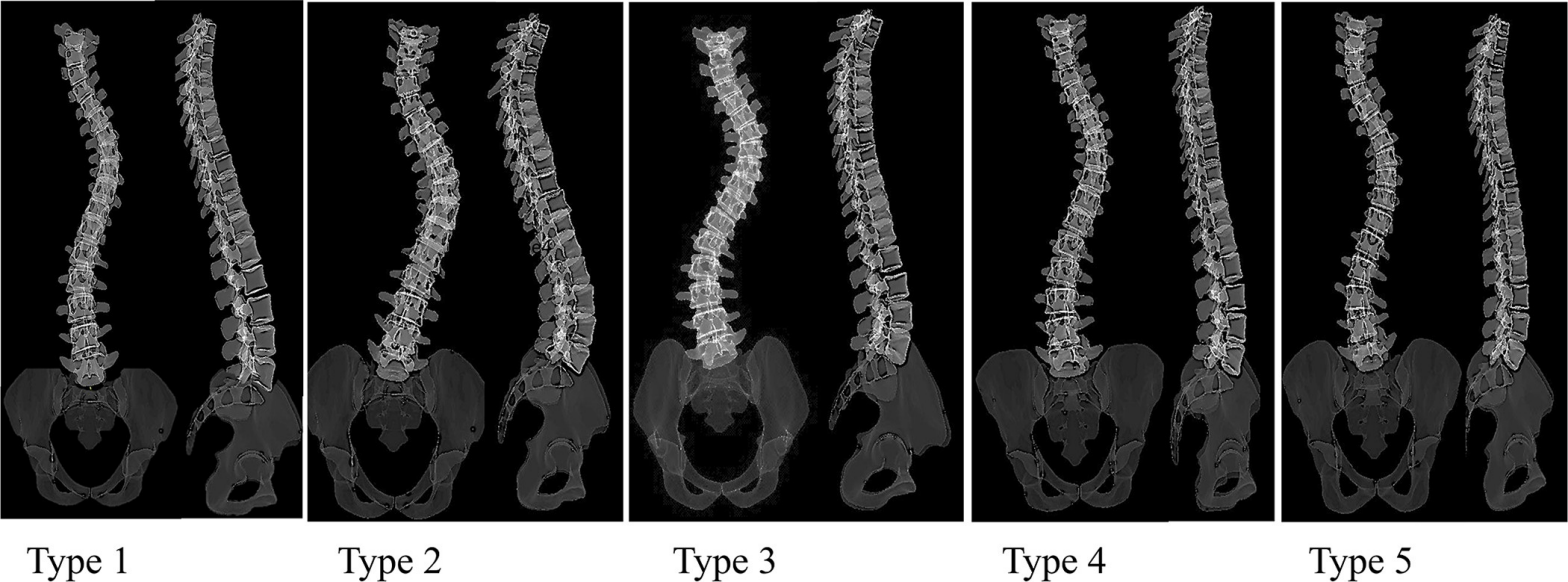
Example of each curve types in frontal and sagittal views.

At a lower Ward’s distance, the two new clusters resulting from dividing the G_1.2_, G_1.2.1_ and G_1.2.2_ subgroups (Fig 2), were not visually different from the other identified subgroups (Supplementary material-S1 Fig). Thus the total number of distinct clusters in the cohort was determined to be five (Type 1- Type 5, Fig 3). The frontal, sagittal and 3D curves of these 5 clusters are superimposed in Fig 4. Fig 5 shows a model example of each of the five clusters.

The distributions of the Lenke types, kyphosis and lumbar modifiers across the final 5 clusters are summarized in Table 2. The number of patients with kyphosis modifier (-) was significantly higher in Type 4 (G_2.2.1_) compared to Type 1 (G_1.1_) and the number of patients with kyphosis modifier (N) was lower in cluster Type 4 compared to Type 1 determined by Chi squared test, p = 0.038 (Table 2). The number of patients with a lumbar modifier C was significantly higher in Type 3 (G_2.1_) compared to Type 2 (G_1.2_), *p*<0.05 (Table 2). Finally, 8%, 7%, 33%, 12% of clusters G_1.2_, G_2.1_, G_2.2.1_, G_2.2.2_ were male. All patients in cluster G_1.1_ were female.

**Table 2:** Distribution of the Lenke types, kyphosis modifiers, and lumbar modifiers across the 5 clusters.

Clusters	Lenke Type		Kyphosis Modifier			Lumbar Modifiers		
	Lenke1	Lenke 2	-	N	+	A	B	C
**Type 1**	74%	26%	21%	55%	24%	62%	20%	18%
**Type 2**	95%	5%	36%	56%	18%	67%	23%	10%
**Type 3**	63%	37%	62%	48%	0	0	0	100%
**Type 4**	100%	0	91%	9%	0	0	35%	65%
**Type 5**	77%	23%	85%	15%	0	0	58%	42%

## Discussion

Despite the general consensus on the importance of the 3D considerations of the spinal deformities in the AIS patients, AIS curve classification for patient monitoring and surgical decision-making remains to be based on the 2D parameters of the spine. The 3D classification of the scoliotic curve has been hampered by lack of clinically accessible 3D parameters that can capture subtle differences between the curve patterns in AIS. To address this shortcoming, we proposed and executed a 3D classification of the spinal curve and described the differences in the pairs of the frontal and sagittal profiles of each cluster that can be identified from the 2D X-rays in a subgroup of 103 AIS patients with a right thoracic curve. Our findings are: 1- a total number of 5 different 3D curve types were identified in right thoracic AIS. 2- the highest level of dissimilarity for 3D classification of the AIS patients with a right thoracic curve is in their sagittal curve type; hypo-thoracolumbar kyphotic (44%) and flat sagittal profile (56%). 3- Two subgroups of AIS with frontal imbalance to the right (Type 2:21%) and to the left (Type 3:11%) were determined, however neither group had a sagittal imbalance. 4- One AIS subgroup with normal sagittal profile and frontal balance was identified, a total of 24% of cohort. 5- The value of the axial representation of the curve to differentiate between different curve types was underlined (Fig 3).

An association between the spinal deformities in the three anatomical planes in the AIS patient’ subgroups has been shown [24]. Since the 3D alignment of the spine impacts both postural balance and the biomechanics of the spine [24, 25], a 3D classification of the spinal deformities can have significant implications in patient monitoring and surgical planning [16]. The 3D classifications of the spine have tried to address the current shortcomings of the 2D classification methods [24, 26–30]. Several 3D parameters describing the mechanical and geometrical properties of the spinal curve have been developed [24, 26–30]. Doung et al. used Fuzzy clustering in a cohort of all Lenke types AIS patients and 11 subtypes were determined [8]. Another study by Sangole et al. used clinical variables of the spinal deformities in main thoracic AIS curve type and determined three subgroups of AIS with different in the axial rotation of the thoracic curve [6]. Kadoury et al. used a dimension reduction technique along with a clustering method in Lenke 1 AIS patients [7]. Their result determined four subgroups in Lenke 1 AIS with different sagittal curve characteristics (1) normal kyphosis/hyper-lordosis, (2) small kyphosis/ normal lordosis, (3) hypo-kyphotic/ hyper-lordosis and (4) hyper-kyphotic (7). Different from Kadoury et al. [7], our analysis yielded in 5 sagittal groups (Figs 3 and 4), one with normal sagittal profile. However, similar to Kadoury’s classification [7] a subgroup with small kyphosis/normal lordosis (G_2.2.2_) was identified. Our subgroups’ sagittal profiles were: (G_1.1_) normal sagittal profile, (G_1.2_) hypo-thoracolumbar kyphosis without a proximal kyphosis, (G_2.1_) hypo-thoracolumbar kyphosis with a proximal kyphosis, (G_2.2.1_) flat sagittal profile without a proximal kyphosis and a high inflection point and finally (G_2.2.2_) flat sagittal profile with a proximal kyphosis and a low inflection point. In addition to the differences in the methodology, differences in the cohort, right thoracic AIS in the current study versus Lenke 1 in Kadoury et al. [7], could have contributed to the differences in the sagittal groups. In another sagittal classification of the spine in all AIS types, 3 subgroups, group 1 with three harmonious sagittal curves, group 2 hypokyphotic, and group 3 thoracolumbar lordotic were identified [31]. Although the cervical spine was not included in our study, the three AIS sagittal types as reported in the previous study [31] could correspond to group 1: G_1.1_, group 2: G_2.2.1_, G_2.2.2_ and group 3: G_1.2_ and G _2.1_ subgroups in the current analysis. Our analysis, however, showed subtle difference in the sagittal profiles, as well as the axial view, between G_2.2.1_ (Type 4) and G_2.2.2_ (Type 5) as well as between G_1.2_ (Type 2) and G_2.1_ (Type 3) (Fig 3). As our study focused on a 3D classification of the spine as opposed to a sagittal classification, the importance of the additional sagittal subgroups as determined in our study in clinical and surgical assessment of the AIS patients remain to be determined. Considering the link between the spinal profile and surgical outcome in AIS, [3, 4, 10, 31, 32] detailing the differences in the 3D profiles, as shown in Fig 3, can play an important role in surgical planning. The impact of the surgeon modifiable factors on the surgical outcomes in each of clusters will be detailed in future studies.

**Fig 6:**
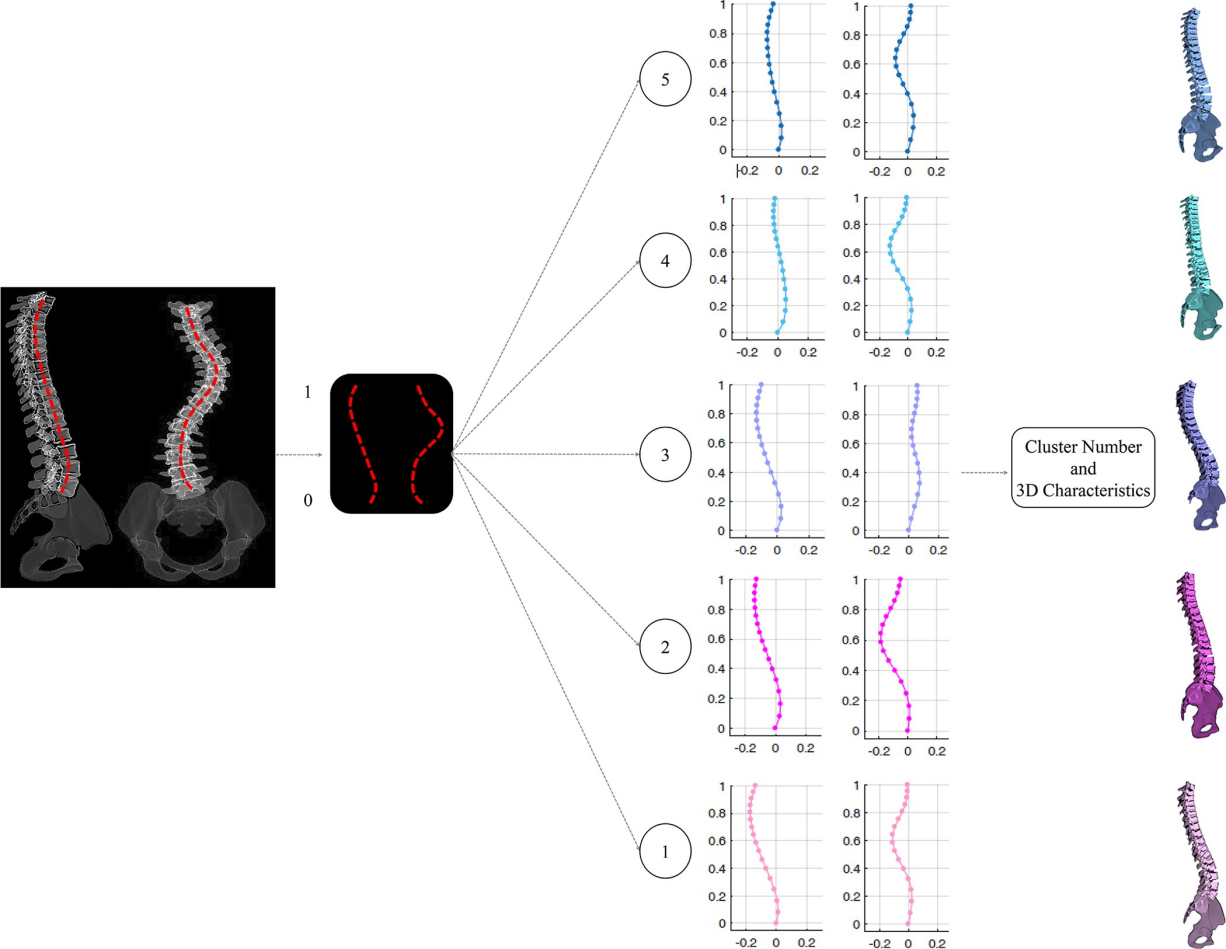
Schematic the proposed application of the 3D classification using only radiographic (2D) images. The frontal and sagittal curves were identified and matched to the current classification subtypes 2D views to determine the 3D classification of the patient from the 2D X-rays.

Our result, once again, showed the importance of the 3D classification in AIS. While considering only frontal (Fig 4A) or sagittal (Fig 4B) views of the spine may suggest grouping of the patients in a manner different from what was shown in Fig 2 and Fig 3, only the 3D spinal curve (Fig 4C) can depict the differences between the G_1_’s (G_1.1_, G_1.2_) and G_2_’s (G_2.1_, G_2.2.1_, G_2.2.2_) subgroups (pinks and blues). Different from other classification methods in which the link between the frontal and sagittal curves are not well defined [33], our 3D classification could characterize pairs of frontal and sagittal spinal curves resulted from a true 3D classification of the curve as they related to different axial view projection of the spine (Fig 3). The pairs of frontal and sagittal curve types have the potential to be used as a method for 3D classification of the curve by matching the patients’ biplanar X-rays and the 2D projections of the 3D clusters (Fig 6). The current advancement in artificial intelligent and deep learning allows extracting anatomical landmarks from X-ray images and matching the frontal and sagittal spinal curve to the closest 3D cluster (Fig 6). A more standardized classification method, which eliminates the inter- and intra-observer reliability, is promised using this method.

In Summary, as the impact of the surgical maneuver on the surgical outcomes in presence of different pre-operative spinal parameters has been shown [16, 31, 32, 34, 35], it is expected that a detailed pre-operative classification of the spine improve the outcome prediction for each subtypes. While the 3D spinal curves were used in our classification, we emphasized on characterizing the pairs of the sagittal and frontal curves as they related to a true 3D classification of the spine (Fig 5), thus bypassing a need for 3D modeling of the spine and facilitating the application of our proposed classification in clinics. Limitations of the study includes a small sample size for the control group however the sagittal measurement of the spine and pelvis of our non-scoliotic cohort matched the normative values of the spine in a cohort of 320 non-scoliotic age-matched adolescent as reported previously [33, 36]. The study focused on only one curve type with a deformity severity at the lower end of surgical range and the classification of the other curve types and more severe curves using a similar approach will be explored in the future.

## Supporting information

S1 Fig The two subgroups of G_1.2_ cluster (G_1.2.1_ and G_1.2.2_) in the three anatomical planes are shown. (DOCX)
