# Supplementary material for: A hierarchical classification of adolescent idiopathic scoliosis: Identifying the distinguishing features in 3D spinal deformities
Source: PLoS One. 2019 Mar 20;14(3):e0213406. doi: 10.1371/journal.pone.0213406 (PMC6426223; doi:10.1371/journal.pone.0213406)

**Supplementary Material**

**A) Additional subgroups**

S1 Fig-The dendrogram (Figure 2) showed that at a Ward’s distance of 790 two branches (G_1.2.1_ and G_1.2.2_) merge and create G_1.2_ cluster. These two clusters, G_1.2.1_ and G_1.2.2,_ are shown below. Different from the other clusters (G_1.1_, G_1.2_, G_2.1_, G_2.2.1_, G_2.2.2_) that showed differences in at least two of three anatomical planes (Figure 3), these two clusters only differ in the sagittal view. G_1.2.1_ sagittal profile resembled the sagittal profile of G_1.1_ whereas G_1.2.2_ was similar to the one in G_1.2_. Considering these differences the clusters G_1.2.1_ and G_1.2.2_ were not considered the main subtypes of the cohort.


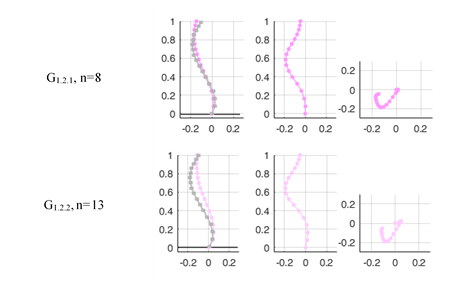

Supplement: S1 Fig — (DOCX) [file pone.0213406.s001.docx]
